# Implantation of Implantable Cardioverter Defibrillators in Kazakhstan

**DOI:** 10.5334/gh.1119

**Published:** 2022-05-10

**Authors:** Temirkhan Begisbayev, Lyazzat Kosherbayeva, Valikhan Akhmetov, Dmitry Khvan, Marzhan Brimzhanova

**Affiliations:** 1 Kazakhstans Medical University “KSPH”, Almaty, Kazakhstan; 2 Asfendiyarov Kazakh National Medical University, Almaty, Kazakhstan; 3 Al-Farabi Kazakh National University, Almaty, Kazakhstan; 4 National Medical Research Center named after Academician E. Meshalkin, Ministry of Health of the Russian Federation, Kazakhstan

**Keywords:** implantable cardioverter defibrillator, sudden cardiac death, subcutaneous implantable cardiac defibrillator, Kazakhstan

## Abstract

**Background and Objectives:**

Implantation of implantable cardioverter-defibrillators (ICD) has increased significantly over the past decade. However, limited data exist regarding practices and policies of ICD implantations in Kazakhstan. We aimed to provide an overview of the current use of ICD in Kazakhstan.

**Methods:**

Using the Unified Healthcare Information System database of the entire Kazakh adult population, statistical and cost data of ICD implantations in 2017–2019 were evaluated. Cardiologists and electrophysiologists working in cardio surgery centers and departments were asked to go through an online survey focused on subcutaneous-ICD (S-ICD) experience.

**Results:**

Implantation of traditional transvenous cardioverter-defibrillators for residents of Kazakhstan is fully reimbursed. A total of 2,263 ICD interventions (2,252 new implantations and 11 reimplantations) were performed across the country during the study period. According to the tariffs approved by the Ministry of Health, the reimbursement cost for one ICD case is about 14,061.80 US dollars. The survey showed that only two hospitals have implanted S-ICDs. Among the main reasons why S-ICD is not widely used in the country the following were named: lack of trained staff (61.1% of respondents); the cost of device and lack of reimbursement (38.7%); and lack of pacing function (27.8%).

**Conclusion:**

The number of ICD implantation in Kazakhstan is steadily continuing to grow, although, compared to developed countries, the implantation rate especially for S-ICD remains low. There is a need in deliberate strategies to remove policy barriers for implementation the most innovative cardiac implantable electronic devices implantations such as S-ICD in the country.

## Introduction

The implantable cardioverter defibrillator (ICD) is an important treatment option for selected patients who have survived cardiac arrest (secondary prevention) or in patients at high risk of sudden cardiac death (SCD) (primary prevention). Randomized trials have consistently shown the effectiveness and efficiency of an ICD in treating cardiac arrhythmias and reducing cardiac mortality [1,2,3]. Recommendations on the use of the ICD have been provided in different important guidelines [4,5]. The cost-effectiveness of ICD therapy has been established in multiple healthcare systems [6,7,8].

Since introduction the ICD devices have undergone further development targeting reduction of size, accumulator longevity, MRI compatibility, remote monitoring, overcoming device-associated complications and improvement patients’ health associated quality of life. A subcutaneous implantable cardiac defibrillator (S-ICD) represents the next step in the evolution of defibrillation technology. S-ICD is known as a safe and effective alternative in appropriate patients and can reduce lead-related complications [9]. The American Heart Association/American College of Cardiology/Heart Rhythm Society (AHA/ACC/HRS) and European Society of Cardiology (ESC) Guidelines include recommendations for using S-ICD as a prevention of SCD [4,5]. Despite the strong evidence base, utilization of ICD therapy, both conventional transvenous and subcutaneous, remains respectively low globally for different reasons.

In Kazakhstan, the State Program has been implemented over the past decades, with priority for strengthening the cardiological care [10,11]. Our study aimed to give an overview of current Kazakh practice regarding conventional ICD and S-ICD implantation.

## Methods

The electronic database from the Unified Healthcare Information System within the Guaranteed Volume of Free Medical Care (GVFMC) and the Compulsory Social Medical Insurance system (CSMI) for the period of 2017–2020 was analyzed retrospectively. The dataset included records on adult patients who underwent ICD surgery (new implantation or generator and electrode replacement) such as geographic region, a medical institution where intervention was held, patients’ demographic characteristics and primary diagnose according to the International Classification of Disease 10th revision (ICD-10).

Information related to the ICD therapy costs were searched from the open sources.

A survey using the Google Form tool was conducted among professionals who perform ICD surgeries. This survey aimed to study standards and policies concerning patients’ management, indications, and techniques of implanting S-ICD in cardiological centers in Kazakhstan. The original questionnaire developed by Serge Boveda et al [12]. (with the author’s permission) was adapted and translated into Russian and Kazakh languages. The questionnaire consisted of 16 questions including standards of care and policies used for patient management, indications, and techniques of implantation of the S-ICDs. A questionnaire was sent via the internet to centers carrying out. The survey was conducted in December 2020. Representatives of 18 from 28 medical centers providing ICD surgery (64.2%) attended the survey.

## Results

During the study period, a total of 2,263 ICD procedures were performed in the Republic of Kazakhstan by 28 medical centers and hospitals. The procedure was a de novo implantation in 2,252 (99.5%) patients and a replacement of a previous ICD system in 11 (0.5%) patients. The annual number of ICD interventions has steadily increased during 2017–2019. The analysis showed that patients from all the 17 regions of Kazakhstan (14 oblasts, Shymkent, Almaty and Nur-Sultan cities) have had access to the intervention.

ICD interventions during the study period prevailed in men by 3 times (78.5%) compared to women. The mean age of patients underwent implantation of ICD slightly increased from 58.6 ± 11 years in 2017 to 59.5 ± 10.7 years in 2019. There were no significant differences in age between genders. One-third of ICD implantation cases were associated with emergency care (27.9%) while other two-thirds were planned admissions. The average length of hospital stay was 10 (±4.9) days. Most of the patients who went through ICD had ischemic cardiomyopathy (ICD-10 I25.5) and dilated cardiomyopathy (ICD-10 I42.2) as main diagnosis (Table 1).

**Table 1:** Baseline characteristics of patients undergoing implantation of ICD.


VARIABLES	2017	2018	2019

Number	719	738	806

New implantations	717 (99.7)	735 (99.6)	800 (99.3)

Replacements	2 (0.4)	3 (0.4)	6 (0.7)

Mean age	58.6 (±11.0)	59,3 (±10.5)	59,5 (±10.7)

Male	577 (80.3)	567 (76.8)	633 (78.5)

Indications			

Primary prevention	506 (70.4)	529 (71.7)	596 (73.9)

Secondary prevention	213 (29.6)	209 (28.3)	210 (26.1)

Mean length of hospital stay	10 (±4.3)	10 (±5.3)	9 (±5.0)

Diagnosis (ICD-10)			

I02.0 Unstable angina	28 (3.9)	25 (3.4)	12 (1.5)

I20.8 Other forms of angina	63 (8.8)	40 (5.4)	9 (1.1)

I25.5 Ischemic cardiomyopathy	195 (27.1)	192 (26.0)	216 (26.8)

I42.0 Dilated cardiomyopathy	182 (25.3)	154 (20.9)	133 (16.5)

I42.2 Other hypertrophic cardiomyopathy	17 (2.4)	9 (1.2)	9 (1.1)

I46.0 Cardiac arrest with successful cardiac recovery	15 (2.1)	9 (1.2)	10 (1.2)

I47.2 Ventricular tachycardia	53 (7.4)	54 (7.3)	65 (8.1)

I48 Atrial fibrillation and flutter	10 (1.4)	19 (2.6)	16 (2.0)

I50.0 Congestive heart failure	34 (4.7)	65 (8.8)	185 (23.0)

I50.1 Left ventricular failure	28 (3.9)	57 (7.7)	49 (6.1)

Other (less than 10 cases each)	94 (13.1)	114 (15.4)	102 (12.7)


Policy for the procedure is guided by national clinical and intervention protocols approved by the Joint Committee for Medical Care Quality of the Ministry of health and available on the Internet. The reimbursement rate for one new implantation of traditional (transvenous) ICD is 14,061.80 US dollar (exchange rate effective by January 2021 is 421,1 KZT per US dollar). The tariffs for replacement are 3,573.20 US dollar and 1,301.30 US dollars for pulse generator and electrode replacement, respectively. The reimbursement cost data were retrieved from the Order of the Minister of Health of the Republic of Kazakhstan ‘On approval of tariffs for medical services provided within the guaranteed volume of free medical care and system of compulsory social health insurance.’

Currently implantation of S-ICD is not covered neither by the GVFMC package neither by CSMI scheme. In 2017 the technology underwent Health Technology Assessment (HTA) but was not entered the country’s reimbursement list. According to the HTA report published by the Republican Center for Health Development of the Ministry of Health of the Republic of Kazakhstan on their website (http://www.rcrz.kz/), it is expected that the cost of S-ICD is four times higher than conventional ICD.

### Results of the survey

Total 36 respondents from 18 cardiac surgery centers or cardiac surgery departments of multidisciplinary hospitals (64%) took part in the survey with a wide geographical distribution of respondents: 3 institutions in Nur-Sultan, 2 in Almaty, 2 in Zhambyl oblast and 11 in other regions. The analysis of the respondents’ answers showed that 11 (61.1%) centers/departments had implanted 50 and less ICD devices, 16.7% had implanted 50–99 ICDs, 16.7% had implanted 100–199 ICDs and only two centers (5.6%) in Almaty and Nur-Sultan reported about 200–300 ICD implantations during the last year. About implantation of S-ICD reported only two representatives of two clinics in Almaty, Nur-Sultan and the number of patients equipped with S-ICD during the last 12 months at their centers were less than 10 and less than 10% of all ICD implantations per center. However, both respondents believe that the volume of patients equipped with S-ICD in the next two years will increase by more than 20% or at least will remain the same. According to them the features favoring S-ICD over traditional ICD the availability of the new generation S-ICD (smaller, MRI compatible, remote monitoring and young age of patients) while the factors that can become an obstacle to the use of S-ICD were patient-related factors such as body size and weight. Both clinics implanting S-ICD have no policy for the procedure. All the S-ICD implantations were performed in EP Laboratory/Coronary angiography laboratory by cardiologist/EP under general anesthesia. The incision strategy was two incisions set (left latero-thoracic + xiphoidal), and ventricular detection screening was performed just before the operation. In one center S-ICD implantation procedures were performed during a short hospitalization (two days) while the second reported about peri-procedural hospitalization for 3–5 days.

Among the reasons reported by the respondents for the nonuse of S-ICD were lack of training (61.1%), economic barriers, such as cost of the procedure (38.7%), and lack of reimbursement (27.8%), lack of pacing function (27.8%) and non-availability of the device (16.7%). Other listed in the questionnaire obstacles to S-ICD use, such as issues associated with patient selection, the absence of eligible patients, patients’ choice, physicians’ skepticism towards device efficacy, or the complexity of the procedure were not selected.

## Discussion

To the best authors’ knowledge, this study is the first attempt to give an overview of the current use of ICD surgery in Kazakh population using a nationwide electronic database. The major findings of this analysis were as follows: 1) the numbers of new implantations and replacements of ICD in Kazakhstan is slowly but steadily increasing; 2) ICD interventions prevail in men and mean age of ICD patients is 59 (±10,7) years; 3) up to 28 cardiac surgery centers and departments across the country provide ICD implantations for patients who is suffered from cardiovascular disease such as ischemic cardiomyopathy (ICD-10 I25.5) and dilated cardiomyopathy (ICD-10 I42.2); 4) implantation of traditional transvenous ICD is included in the reimbursement list and current medical tariff 14,061.80 US per hospitalization case, while the more innovative and more expensive (~4 times higher in comparison with conventional ICD) S-ICD is not covered by the GVFMC and CSMI packages; 5) during the last year S-ICD were implanted to less than in 10 patients and the reasons why S-ICD is not widely used are lack of training and economic barriers (high cost and lack of reimbursement).

Although the authors found that the ICD implantation in Kazakhstan has trend of increase, this implantation rate is still quite low compared to other countries [13]. In 2019 in Kazakhstan the implantation rate of ICD was 4.3 per 100,000 populations. The annual ICD implantation rate of US and Western Europe 11 times (46.2 in 2006) [14] and 6 times (25.5 in 2014) [15] higher than that of Kazakhstan in 2019. However, the annual new implantation rate of Kazakhstan in 2017 (3.9 per 100,000) was two higher than that of Korea times in 2016 (1.9 per 100,000) [13]. In Spain, 172 hospitals implanted ICD in 2019 and the total number of registered implantations was 14.9 per 100,000 inhabitants [16].

The average age of patients who received ICD ranged 50–70 years [17], similar to the results of our study (59 years), however recent various studies show that the numbers of patients receiving an ICD up to 70 years have trend to increase [13,18], especially in male patients when compared to female [18,19]. We found that ischemic cardiomyopathy was the most common cardiac diagnosis among ICD patients, the same results indicated Vivienne A Ezzat et al [20]. in a systematic review and Chao T. et al [21]. in Taiwan experience. However, since the electronic database analyzed in this study contained only information on the codes of the main diagnosis and intervention underwent the authors were not able to assess the rationality of using ICD therapy from the evidence base point of view.

Serge Boveda et al [8]. reported that in European countries the most important barrier to the implementation of S-ICD were high costs and lack of reimbursement (25%), while in Kazakhstan the main reason named lack of trained specialists (61.1%).

This study has some limitations. First, the study lacks any reference to the prevalence of cardiac conditions and left ventricular ejection fraction which is well known to be a fundamental patient selection criterion, especially in primary prevention for the indication for ICD implantation. The authors had a limited set of data. However, this is the first attempt to describe the current practice regarding ICD therapy in the country and may be useful for researchers. More in-depth analysis of the medical records of patients who received the intervention is required to give a more accurate picture of Kazakh clinical practice for ICD therapy.

The representatives of cardiology centers attended voluntarily, so not all organizations attend the survey.

## Conclusion

Based the study results and because the life expectancy of the population of the Republic of Kazakhstan is slowly but steadily growing it is expected that cardiac implantable electronic devices implantations such as ICD procedures will continue to increase. Consequently, higher economic burden for device therapy is expected in the future. However, compared to Western developed countries the new implantation rate of ICD in Kazakhstan remains low. Well-designed and comprehensive strategies to improve underutilization of ICD, especially the most modern and improved technologies such as S-ICD are required to improve cardiac care and sudden cardiac death prophylaxis in Kazakhstan.

## References

[1] Barra S, Providência R, Tang A, Heck P, Virdee M, Agarwal S. Importance of Implantable Cardioverter-Defibrillator Back-Up in Cardiac Resynchronization Therapy Recipients: A Systematic Review and Meta-Analysis. J Am Heart Assoc. 2015; 4(11): e002539. Published 2015 Nov 6. DOI: 10.1161/JAHA.115.002539 26546574 PMC4845241

[2] Akel T, Lafferty J. Implantable cardioverter defibrillators for primary prevention in patients with nonischemic cardiomyopathy: A systematic review and meta-analysis. Cardiovasc Ther. 2017; 35: e12253. DOI: 10.1111/1755-5922.12253 28129469

[3] Shun-Shin MJ, Zheng SL, Cole GD, Howard JP, Whinnett ZI, Francis DP. Implantable cardioverter defibrillators for primary prevention of death in left ventricular dysfunction with and without ischaemic heart disease: a meta-analysis of 8567 patients in the 11 trials. Eur Heart J. 2017; 38(22): 1738–1746. DOI: 10.1093/eurheartj/ehx028 28329280 PMC5461475

[4] Al-Khatib SM, Stevenson WG, Ackerman MJ, et al. AHA/ACC/HRS guideline for management of patients with ventricular arrhythmias and the prevention of sudden cardiac death: a report of the American College of Cardiology/American Heart Association Task Force on Clinical Practice Guidelines and the Heart Rhythm Society. J. Am. Coll. Cardiol; 2017. 10.1016/j.jacc.2017.10.054 29097296

[5] Priori SG, Blomström-Lundqvist C, Mazzanti A, et al. Task Force for the Management of Patients with Ventricular Arrhythmias and the Prevention of Sudden Cardiac Death of the European Society of Cardiology (ESC), 2015 ESC Guidelines for the management of patients with ventricular arrhythmias and the prevention of sudden cardiac death: the Task Force for the Management of Patients with Ventricular Arrhythmias and the Prevention of Sudden Cardiac Death of the European Society of Cardiology (ESC) endorsed by: Association for European Paediatric and Congenital Cardiology (AEPC). Europace. 17 2015; 1601–1687. 26318695 10.1093/europace/euv319

[6] Higuera L, Holbrook R, Wherry K, et al. Comparison of cost effectiveness of implantable cardioverter defibrillator therapy in patients for primary prevention in Latin America: an analysis using the Improve SC; 2021. DOI: 10.1080/13696998.2021.1877451 33471579

[7] Holbrook R, Higuera L, Wherry K, et al. Implantable cardioverter defibrillator therapy is cost effective for primary prevention patients in Taiwan: An analysis from the Improve SCA trial. PLoS ONE. 2020; 15(11): e0241697. DOI: 10.1371/journal.pone.0241697 33211698 PMC7676667

[8] Boriani G, Biffi M, Martignani C, Gallina M, Branzi A. Cost-effectiveness of implantable cardioverter-defibrillators. European Heart Journal. 2001; 22(12): 990–996. DOI: 10.1053/euhj.2000.2371 11428834

[9] Basu-Ray I, Liu J, Jia X, et al. Subcutaneous Versus Transvenous Implantable Defibrillator Therapy: A Meta-Analysis of Case-Control Studies. JACC Clin Electrophysiol. 2017 Dec 26; 3(13): 1475–1483. DOI: 10.1016/j.jacep.2017.07.017 29759827

[10] Government of the Republic of Kazakhstan. State Healthcare Development Program, ‘Densaulyk’ for 2016–2019. Approved Government Decree No. 634. Astana: October 15, 2018. http://adilet.zan.kz/rus/docs/P1800000634. (Accessed 13 January 2022).

[11] Kulzhanov M, Rechel B. Kazakhstan: health system review. Copenhagen: World Health Organization. Regional Office for Europe. 2007. https://apps.who.int/iris/handle/10665/107868. (Accessed 13 January 2022).

[12] Boveda S, Lenarczyk R, Haugaa K, et al. Implantation of subcutaneous implantable cardioverter defibrillators in Europe: results of the European Heart Rhythm Association survey. Europace. 2016 Sep; 18(9): 1434–9. DOI: 10.1093/europace/euw258 27582309

[13] Lee JH, Lee SR, Choi EK, et al. Temporal Trends of Cardiac Implantable Electronic Device Implantations: a Nationwide Population-based Study. Korean Circ J. 2019 Sep; 49(9): 841–852. DOI: 10.4070/kcj.2018.0444 31074230 PMC6713826

[14] Greenspon AJ, Patel JD, Lau E, et al. 16-year trends in the infection burden for pacemakers and implantable cardioverter-defibrillators in the United States 1993 to 2008. J Am Coll Cardiol. 2011 Aug 30; 58(10): 1001–6. DOI: 10.1016/j.jacc.2011.04.033 21867833

[15] Pekka Raatikainen MJ, Arnar DO, Zeppenfeld K, Jose Luis Merino JL, Kuck K-H, Hindricks G. Current trends in the use of cardiac implantable electronic devices and interventional electrophysiological procedures in the European Society of Cardiology member countries: 2015 report from the European Heart Rhythm Association. EP Europace. 2015 August; 17(4): iv1–iv72. DOI: 10.1093/europace/euv265 26286028

[16] Lozano IF, Asensi JO, Rodríguez JA, Spanish Implantable Cardioverter-defibrillator Registry. 16th Official Report of the Heart Rhythm Association of the Spanish Society of Cardiology (2019). Rev Esp Cardiol. 2020; 73(12): 1026–1037. https://www.revespcardiol.org/en-spanish-implantable-cardioverter-defibrillator-registry-16th-articulo-S1885585720304011. DOI: 10.1016/j.rec.2020.07.015 33039380

[17] Barra S, Providência R, Paiva L, Heck P, Agarwal S. Implantable cardioverter-defibrillators in the elderly: rationale and specific age-related considerations. Europace. 2015 Feb; 17(2): 174–86. DOI: 10.1093/europace/euu296 25480942

[18] Lin G, Meverden RA, Hodge DO, Uslan DZ, Hayes DL, Brady PA. Age and gender trends in implantable cardioverter defibrillator utilization: a population based study. J Interv Card Electrophysiol. 2008; 22(1): 65–70. DOI: 10.1007/s10840-008-9213-6 18324458 PMC2719248

[19] Foo FS, Lee M, Looi K-L, et al.; on behalf of the ANZACS-QI investigators. Implantable cardioverter defibrillator and cardiac resynchronization therapy use in New Zealand (ANZACS-QI 33). J Arrhythmia. 2020; 36: 153–163. DOI: 10.1002/joa3.12244 PMC7011834 32071634

[20] Ezzat VA, Lee V, Ahsan S, et al. A systematic review of ICD complications in randomised controlled trials versus registries: is our ‘real-world’ data an underestimation? Open Heart. 2015; 2(1): e000198. Published 2015 Feb 17. DOI: 10.1136/openhrt-2014-000198 25745566 PMC4346580

[21] Chao TF, Lai CH, Tuan TC, et al. Long-Term Prognosis in Recipients of Implantable Cardioverter-Defibrillators for Secondary Preventions in Taiwan – A Multicenter Registry Study. Acta Cardiol Sin. 2014; 30(1): 22–28. 27122764 PMC4804817

